# Case report: Successful autologous hematopoietic stem cell transplantation in a patient with GAD antibody-spectrum disorder with rapidly progressive dementia

**DOI:** 10.3389/fneur.2023.1254981

**Published:** 2023-10-19

**Authors:** Caio César Diniz Disserol, Dora Pedroso Kowacs, Samir Kanaan Nabhan, Hélio Afonso Ghizoni Teive, Pedro André Kowacs

**Affiliations:** ^1^Department of Neurology, Instituto de Neurologia de Curitiba, Curitiba, Brazil; ^2^Department of Neurology, Complexo do Hospital de Clínicas da Universidade Federal do Paraná, Curitiba, Brazil; ^3^Blood and Marrow Transplantation Program, Hospital de Clínicas, Federal University of Paraná, Curitiba, Brazil

**Keywords:** autoimmune diseases of the nervous system, dementia, encephalitis, glutamic acid decarboxylase, neurocognitive disorders

## Abstract

The prevalence of neurological syndromes associated with antibodies to glutamic acid decarboxylase is increasing. While cognitive impairment is a common feature of this condition, it seldom emerges as the primary symptom. In this study, we discuss a case of refractory dementia associated with the glutamic acid decarboxylase spectrum disorder. Interestingly, this case showed a favorable outcome following autologous hematopoietic stem cell transplantation. We also provide an in-depth review of the current literature on the use of this therapeutic approach for the treatment of this disease.

## Introduction

The range of neurological syndromes associated with antibodies to glutamic acid decarboxylase (GAD) continues to expand. Documented syndromes encompass stiff-person syndrome (SPS), ataxia, limbic encephalitis, epilepsy, nystagmus, and myoclonus (1). Cognitive impairment frequently appears in association with these syndromes (2), and isolated, rapidly progressive dementia has been observed (3). Collectively, these syndromes are now designated as GAD antibody-spectrum disorders (GAD-SDs) (1).

Treatment of GAD-SDs primarily involves pharmacological interventions to alleviate symptoms, complemented by immunotherapy. The majority of clinical evidence supporting immunotherapy is drawn from studies focused on patients with stiff-person syndrome, as SPS remains the most commonly diagnosed manifestation of GAD-SD. Thus, therapeutic strategies for other GAD-SDs are often derived from these data. The primary immunotherapy employed is intravenous immunoglobulin (IVIg) because of its proven efficacy in SPS. Other immunotherapeutic modalities with variable success include plasmapheresis, corticosteroids, and immunosuppressants. For patients who are resistant to these therapies, hematopoietic stem cell transplantation (HSCT) may be promising (1, 4).

In this paper, we discuss a patient who presented with rapidly progressive dementia and later manifested other GAD-SD symptoms. Despite being resistant to multiple immunotherapies, the patient responded positively to HSCT. Additionally, we provide a review of GAD-SD cases in the literature that have undergone HSCT treatment.

## Case description

A 50-year-old woman, who had been a bank branch manager, sought medical attention in February 2015 due to a recent onset of forgetfulness. Over a period of weeks, she struggled with memorizing passwords and phone numbers, recognizing familiar clients, and performing work tasks. Within 2 months, she was experiencing frequent feelings of déjà vu. These symptoms, although fluctuating, progressively worsened, culminating in spatial disorientation that prevented her from leaving her home without assistance.

The patient’s medical history included regular smoking, hypertension, ischemic heart disease, obstructive sleep apnea, and hypothyroidism. Neurological examinations revealed pronounced memory impairment, executive dysfunction, and visuospatial deficits. Comprehensive neuropsychological evaluations between April and August 2015 confirmed this deterioration (Table 1, assessments A* and B*).

**Table 1:** Comparative table of formal neuropsychological assessments (pre- and post-HSCT).

Test	A* April 2015	B* August 2015	C* March 2023	Comparison* C x A or C x B
	Values in Z score			Difference ≥ 0,5 DP
RAVLT – A1-A5^1^	−0.3	−1.25	−0.7	**Improvement** (C > B)
RAVLT – A6^2^	0	−0.29	−0.2	No change (C = A; C=B)
RAVLT – A7^3^	0	−0.58	−0.5	No change (C = A; C=B)
WMS – LM I^4^	0	−0.3	0.3	**Improvement** (C > B)
WMS – LM II^5^	−0.3	0	0.7	**Improvement** (C > A; C > B)
CFT – Copy^6^	2.5	2.5	2.5	No change (C = A; C=B)
CFT – Immediate^7^	−0.5	0.1	-0.1	No change (C = A; C=B)
CFT^7^ – Delay^8^	0.4	−0.05	-0.05	No change (C = A; C=B)
WAIS-R^9^ – Digit Span	−0.3	−2	−0.6	**Improvement** (C > B)
WAIS-R^6^ – Information	0	0.3	0.3	No change (C = A; C=B)
WAIS-R^6^ - Vocabulary	−0.6	−0.3	−0.3	No change (C = A; C=B)
WAIS-R^6^ - Similarities	0	0.7	0.7	**Improvement** (C > A)
WAIS-R^6^ - Arithmetic	0	−0.3	0.3	**Improvement** (C > A)
WAIS-R^6^ – Pict. Comp.^10^	−1.7	–	0.7	**Improvement** (C > A)
WAIS-R^6^ – Block Design	–	−0.6	−0.3	**Improvement** (C > A)
WAIS-R^6^ – L-N^11^	−0.3	–	1	**Improvement** (C > A)
WAIS-R – Digit Symbol-Coding	−0.3	−2	−0.6	**Improvement** (C > B)
Five-point Test	−1.5	−2	−0.3	**Improvement** (C > A; C > B)

No 2023 test scores decreased in comparison with scores from previous assessments.

^1^RAVLT – A1–A5: Rey Auditory Verbal Learning Test – Learning curve: tests A1 to A5. ^2^RAVLT – A6: immediate recall (after distraction list B1, not presented here). ^3^RAVLT – A7: delayed recall (after 20 min). ^4^WMS-LM I- Wechsler Memory Scale – Logical Memory I subtest: immediate recall. ^5^WMS-LM II: delayed recall. ^6^CFT: Rey-Osterieth Complex Figure Copy: immediate copy for posterior reproduction. ^7^CFT: immediate reproduction (3′ after copying). ^8^CFT: delayed reproduction (30′ after copying). ^9^WAIS-R: Weschsler Adult Intelligence Scale – Revised. ^10^Pict. Comp.: WAIS-R Picture Completion subtest. 11 L-N: WAIS-R Letter-Number subtest.

Initial blood work showed elevated glycated hemoglobin (HbA1c of 8.0%) but a standard metabolic panel, including thyroid function, vitamin B12, homocysteine, and folate levels. Serological tests for HIV, syphilis, and hepatitis were negative, and inflammatory markers were unremarkable. Although brain MRI and 18F FDG-PET scans were normal, EEG detected epileptiform discharges from the left temporal lobe. Investigations for autoimmune encephalopathies revealed significantly raised serum levels of anti-GAD (>2,000 IU/mL) and anti-ZnT8 (>500 IU/mL) antibodies, the latter being linked to type 1 diabetes. CSF analysis was typical, but anti-GAD antibodies were present. Other anti-neuronal antibody tests, both surface and intraneuronal, were negative. Neoplastic screening was unremarkable.

The patient was initially treated with methylprednisolone (1 g daily for 3 days) without improvement. Rituximab was then administered and adjusted based on the CD19 count. Despite a partial response and reduced serum anti-GAD levels, over the next 2 years, the patient developed left temporal lobe epilepsy, diabetes, ataxia, and stiff limb syndrome symptoms in her right leg. Intravenous immunoglobulin (IVIg) treatment was considered but was unavailable due to the COVID-19 pandemic. Azathioprine was tried unsuccessfully.

Recurrent episodes of isolated cognitive decline persisted. They were managed with high-dose corticosteroids, although symptom relief seemed to stem mainly from the adjustment of symptomatic treatments. Three years into azathioprine treatment, the patient suffered a subacute decline in all GAD-SD symptoms that correlated with high serum anti-GAD levels. Azathioprine was halted, and although monthly low-dose IVIg was attempted, higher doses were denied by her health insurance. At this point, autologous hematopoietic stem cell transplantation (HSCT) was proposed.

**Figure 1:**
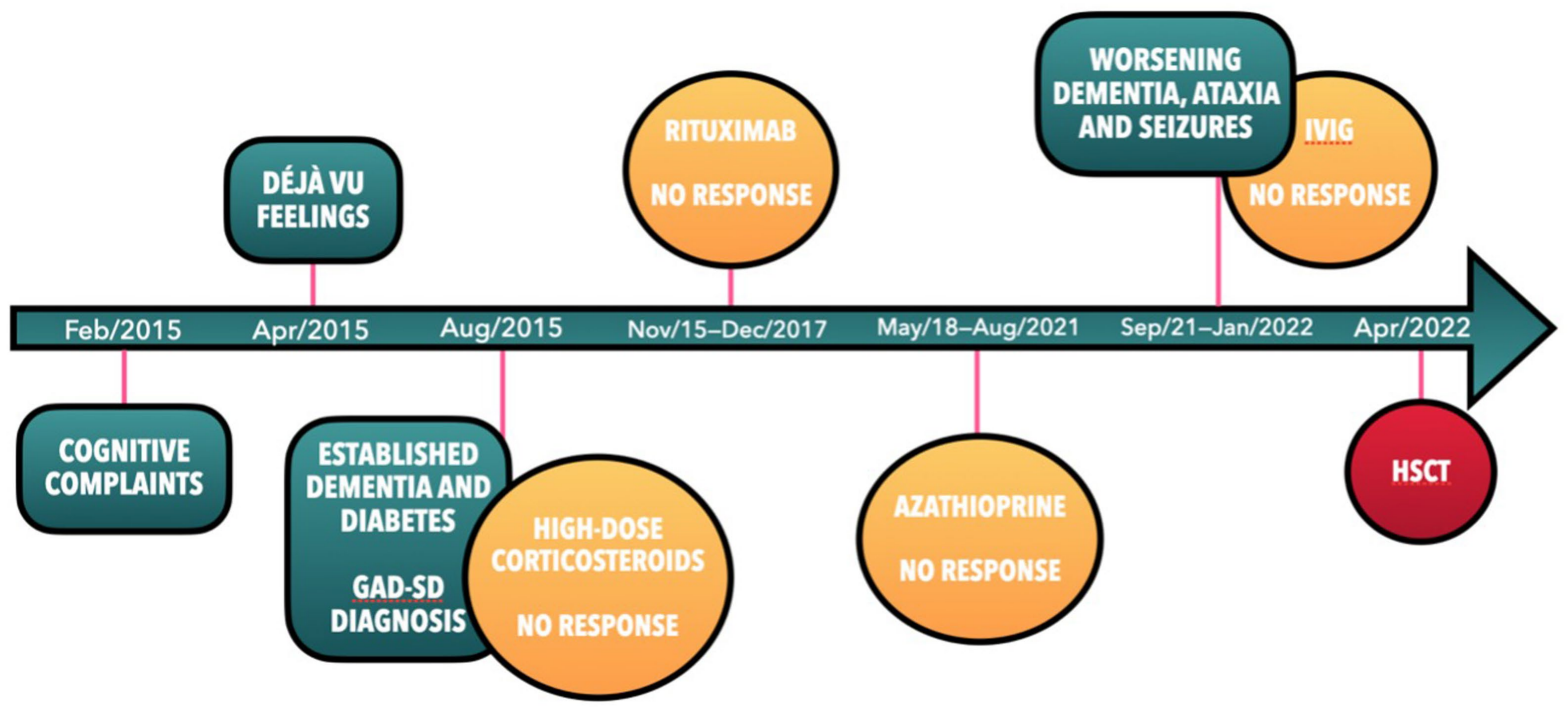
Timeline of key events.

Seven years after her initial symptoms (April 2022), the patient underwent HSCT. Despite post-transplant complications, such as treatment-resistant diarrhea due to pseudomembranous colitis, she displayed improvements in both physical and cognitive function (Figure 1, patient timeline). A follow-up neuropsychological assessment 10 months post-HSCT showed enhanced cognitive performance across various domains (Table 1, assessment C*). Subsequent brain MRIs and EEGs were standard. The patient regained many higher-level functions, managed her banking independently, and achieved better glycemic control, even discontinuing insulin use. Currently, her Modified Rankin Scale (mRs) score is 0, indicating no symptoms.

## Discussion

“Dementia” refers to an acquired cognitive impairment in one or more cognitive domains. This decline from a previous level of functioning interferes with daily life activities and with an individual’s independence (5, 6). While neurodegenerative etiologies account for the majority of dementia cases (7), it is essential to identify potentially treatable causes (8, 9). Autoimmune etiologies should be considered, especially in instances with a rapidly progressive course, fluctuating symptoms, and the presence of seizures (9). It should be noted that these features are not exclusive. For example, rapidly progressive dementia can manifest in various diseases (10). Conditions like Lewy body disease, Parkinson’s disease, and vascular cognitive impairment can exhibit fluctuating symptoms (11). Furthermore, seizures are commonly associated with dementia (12).

Rapidly Progressive Dementia (RPD) constitutes a small fraction of all dementias (3–4%). It is characterized by cognitive and functional impairments that manifest within 1–2 years, often within just weeks or months, as seen in our patient’s initial presentation (13). RPD can have different etiologies, with the most common being prion (a prototypical RPD), autoimmune, infectious, vascular, metabolic, neoplastic, and atypical manifestations of traditional degenerative diseases such as Alzheimer’s disease. Prompt evaluation is vital to identify potential treatable causes, such as autoimmune and inflammatory etiologies (10, 13).

The 1960s saw the first suspected descriptions of cognitive impairment due to autoimmune encephalitis. In 1966, Lord Brain chronicled a patient’s cognitive decline not associated with cancer (14). By 1968, Corsellis and colleagues had defined paraneoplastic limbic encephalitis as a distinct clinicopathological entity (15). Since then, our understanding of autoimmune encephalitis has grown exponentially, leading to the identification of numerous antineuronal antibodies (16), some of which cause dementia. Such cases are occasionally referred to as “autoimmune dementias” or “autoimmune encephalopathies” (9, 17). A study of 75 RPD cases over three years at a tertiary center identified 15 instances of an autoimmune nature, one of which was linked to anti-GAD antibodies (18). In the literature, we identified eight cases of anti-GAD dementia (refer to Table 2).

**Table 2:** Anti-GAD dementia cases.

Author	Year	*n*	Pre-treatment antibody titers	Treatment	Outcomes
Akkari et al. (19)	2021	1		IVIg	Improvement
Mirabelli-Badener et al. (20)	2012	1	Anti-GAD 641 U/mL (serum) Anti-Abs 154 pmol/L	Methylprednisolone + IVIg + Mycophenolate+ Rituximab	No improvement in symptoms. Only reduction in anti-GAD levels (<69 U/mL)
Markakis et al. (21)	2014	1	Anti-GAD 37,550 UI/mL (serum) 15,400UI/mL (liquor)	Methylprednisolone + PLEX+ Prednisolone	Symptom improvement and decrease in anti-GAD serum levels (9,600UI/mL)
Alencar et al. (22)	2017	1	Anti-GAD >100 U/mL (serum)	Methylprednisolone + IVIg + Glatiramer	Symptom improvement
Takagi et al. (23)	2011	1	Anti-GAD 2,865.2 U/mL (serum) (67.8 U/mL)	IVIg	No improvement in symptoms or reduction of antibody levels
Ren et al. (24)	2021	3	Anti-GAD 19,610UI/ml (serum) 3,325UI/mL (CSF) ______________ >300UI/mL (serum) >300 UI/ml (CSF) _______________ 3,400UI/mL (serum) 13UI/mL (CSF)	Methylprednisolone + IVIg + PLEX _____________ Methylprednisolone + IVIg __________ Methylprednisolone + IVIg, Mycofenolato, Rituximabe, PLEX	Symptom improvement ______________ No improvement in symptoms __________________ Symptom improvement

GAD, gamma-aminobutyric acid decarboxylase; *N*, number; IVIg, intravenous immunoglobulin; PLEX, plasma exchange; MP, methylprednisolone; Anti-GAD serum levels, normal, <1 UI/mL – positive, >5 UI/mL – high, > = 2,000 UI/mL; Anti-GAD CSF levels, normal:<1 UI/mL- high:> 100 UI/mL.

Glutamic acid decarboxylase (GAD) is an enzyme predominantly found in the central nervous system (CNS) and pancreatic beta cells. The first identification of autoantibodies targeting GAD dates back to 1988. In subsequent years, GAD antibodies have been linked to other clinical manifestations such as cerebellar ataxia, limbic encephalitis, myoclonus, and nystagmus. These varied clinical syndromes associated with GAD antibodies have been collectively categorized as “GAD antibody-spectrum disorders” (1).

Treatment strategies for GAD-SDs, excluding SPS, have not been universally agreed upon. However, intravenous immunoglobulin (IVIg) is a prominently recognized modality, especially given its demonstrated efficacy in SPS patients (1, 25). The applicability of treatments across the range of GAD-SD manifestations remains an area of uncertainty, but current approaches seem plausible.

Recent literature has highlighted the potential for treating SPS using autologous hematopoietic stem cell transplantation (HSCT) (26–28). Cumulatively, these studies examined 29 patients who underwent HSCT (Table 3). While IVIg is a costly and long-term immunomodulatory strategy, autologous HSCT, despite its inherent risks, holds promise for inducing prolonged remissions not only in GAD-SD but also in other neurological autoimmune disorders (29). Extensive consultations were held with our patient and her family regarding the potential benefits and risks of HSCT. The patient had expressed feelings of disappointment and depression stemming from the relentless progression of her disease and numerous unsuccessful treatments with conventional immunomodulatory and immunosuppressive strategies.

**Table 3:** Autologous HSCT for anti-GAD spectrum disorders.

Author	Year	Condition (*n*)	Antibody titers (serum)	Previous treatment	Outcomes
Sanders et al. (26)	2014	SPS (2)	Anti-GAD: 5.6 and 127 Ui/mL	IVIg + Azathioprine + PLEX	Long-term remission
Kass-Iliyya et al. (27)	2021	SPS (3) PERM (1)	SPS pts.: Anti-GAD >2000 Ui/mL PERM pt.: Anti-GAD 372 Ui/mL + Anti-Gliadin positive + Anti- Glycine positive	IVIg +/− PLEX +/− Rituximab	All patients improved mobility and ambulation
Burt et al. (28)	2021	SPS (23)	Anti-GAD: 2,5 to >250 Ui/mL	IVIg +/− Rituximab or Azathioprine	17 responders (11 in remission for 3.5 years) – improvement in stiffness, spasms, mobility, and quality of life 6 non-responders

HSTC, hematopoietic stem cell transplantation; GAD, gamma-aminobutyric acid decarboxylase; (*n*), number of cases. SPS, stiff person syndrome; PERM, progressive encephalomyelitis with rigidity and myoclonus; EMG, electromyography; Cy, cyclophosphamide; G-CSF, granulocyte colony-stimulating factor; ATG, anti-thymocyte globulin; Anti-GAD 65, immunoprecipitation assay (IPA) / < ou igual a 0.02 nmol/L liquor; Anti-GAD -ELISA- serum level, POSITIVE > 10 UI/ML AND HIGH LEVEL > 100.000.

Autologous HSCT, as previously mentioned, is not without risks. Patients undergoing this procedure face potential threats from opportunistic infections and adverse reactions related to the drugs used (30). Notably, there is a documented case of a patient who developed severe anti-GAD encephalitis following an HSCT procedure (31). Additionally, other autoimmune conditions may emerge post-procedure (32). It is imperative that these considerations be meticulously weighed when recommending autologous HSCT to any patient diagnosed with GAD-SD. Nevertheless, our patient, fully aware of these risks, expressed that she would opt for the same course of treatment if faced with the decision again.

## Conclusion

There is a broad spectrum of neurological conditions that can manifest as rapidly progressive dementia. Among these, autoimmune dementias, such as those presenting as GAD-SD, should always be on the differential list. Accurate diagnosis is pivotal, as it can guide appropriate treatment. In instances where patients are unresponsive to initial immunotherapies, consideration of HSCT as a treatment option becomes crucial.

## Data availability statement

The original contributions presented in the study are included in the article/supplementary material, further inquiries can be directed to the corresponding author.

## Ethics statement

Ethical review and approval was not required for the study on human participants in accordance with the local legislation and institutional requirements. Written informed consent from the patients/participants or patients/participants’ legal guardian/next of kin was not required to participate in this study in accordance with the national legislation and the institutional requirements. Written informed consent was obtained from the individual(s) for the publication of any potentially identifiable images or data included in this article.

## Author contributions

CD: Conceptualization, Writing – original draft, Writing – review & editing. DK: Conceptualization, Writing – original draft, Writing – review & editing. SN: Conceptualization, Writing – review & editing. HT: Conceptualization, Writing – review & editing. PK: Conceptualization, Writing – original draft, Writing – review & editing.
